# Hyperpolarized-MRI in Hypertrophic Cardiomyopathy: A Narrative Review

**DOI:** 10.1177/11795468251369234

**Published:** 2025-08-29

**Authors:** Ali Malik, Sukruth Pradeep Kundur, Sanjay Sivalokanathan

**Affiliations:** 1 Faculty of Life Sciences and Medicine, King’s College London, UK; 2 Mount Sinai Fuster Heart Hospital, Icahn School of Medicine, New York, NY, USA

**Keywords:** hyperpolarized-MRI, hypertrophic cardiomyopathy, inherited cardiac diseases, ^13^C pyruvate

## Abstract

Hypertrophic cardiomyopathy is a genetically inherited cardiac disorder that presents with diverse clinical phenotypes. It is associated with significant adverse outcomes, including arrhythmias and sudden cardiac death. Current gold-standard diagnostic methods include echocardiography and cardiac magnetic resonance imaging. These imaging modalities are the cornerstone in identifying structural abnormalities and aiding risk stratification. However, they fail to capture the preceding cellular and metabolic disturbances that underpin disease progression. Hyperpolarized magnetic resonance imaging (HP-MRI) is an emerging imaging technique that enables non-invasive and non-ionizing visualization of metabolic pathways. HP-MRI enhances the signal of metabolites like [1-^13^C]pyruvate, providing insights into metabolic pathways. Alterations in the metabolic pathways of cardiomyocytes are central to HCM pathophysiology. HP-MRI may be able to delineate the metabolic consequences of sarcomere mutations and distinguish HCM from phenocopies such as glycogen storage disorders or cardiac amyloidosis. More importantly, it has the potential to detect early metabolic shifts and thus play a role in early diagnosis, personalized risk stratification, and monitoring therapeutic response. Although still in experimental stages with technical challenges, HP-MRI has demonstrated considerable potential in preclinical and small-scale studies, exhibiting effectiveness in the diagnosis and monitoring of malignancies across a substantial number of investigations. Further research focusing on larger cohorts and integrating HP-MRI with traditional cardiovascular imaging may pave the way for its clinical use, as well as risk stratification, in HCM.

## Introduction

Hypertrophic cardiomyopathy (HCM) is a complex and heterogeneous cardiac disorder characterized by unexplained myocardial hypertrophy in the absence of secondary causes such as systemic hypertension or aortic stenosis. It is among the most prevalent inherited cardiac conditions, with an estimated prevalence of 0.16% to 0.29%, and affects individuals across all demographic groups.^1^ Clinically, HCM manifests with symptoms such as dyspnea, chest pain, and syncope, driven by mechanisms including left ventricular outflow tract (LVOT) obstruction, mitral regurgitation, myocardial ischemia, and arrhythmias.^2^ Given its variable presentation, genetic basis, and potential for sudden cardiac death (SCD), HCM remains a central focus of cardiology research.^3^

At a molecular level, HCM is primarily caused by mutations in sarcomeric proteins that regulate cardiomyocyte contractility. The most common mutations occur in the MYH7 and MYBPC3 genes.^4^ These mutations disrupt force generation and relaxation dynamics, contributing to hypercontractility, impaired relaxation, and ultimately hypertrophic remodeling. Over time, secondary changes such as myocardial fibrosis and microvascular dysfunction emerge.^5^ More than 1,500 pathogenic variants have been identified, yet only ~40% of patients carry a clearly identifiable mutation.^6^ Accordingly, patients are broadly classified into sarcomeric-positive (Sarc⁺) and sarcomeric-negative (Sarc⁺) subgroups.^7^

Diagnostic pathways have evolved substantially with imaging advances. Echocardiography remains the first-line tool, useful for identifying LVH and dynamic obstruction.^8^ However, its limited sensitivity in early or apical disease has led to the wider use of cardiac magnetic resonance (CMR). CMR offers superior resolution and tissue characterization, aiding in the detection of myocardial fibrosis, valvular abnormalities, and subtle phenotype variants.^9,10^ Late gadolinium enhancement (LGE) on CMR has become a key noninvasive marker of myocardial fibrosis and is used for risk stratification.^11^ CMR also outperforms echocardiography in distinguishing HCM from phenocopies such as cardiac amyloidosis or athlete’s heart.^12^

Yet despite these advances, several critical gaps remain. Current imaging modalities cannot detect metabolic alterations that precede hypertrophy, nor can they capture real-time shifts in cardiac energetics.^13^ Tools such as PET provide insight into glucose uptake but involve ionizing radiation and lack downstream metabolic resolution.^13^ This is especially limiting in patients who are genotype-positive but phenotype-negative, or when evaluating early therapy response.

Hyperpolarized MRI (HP-MRI) represents a novel solution to these limitations. By hyperpolarizing [1-¹³C]pyruvate, this technique enhances MR signal by several orders of magnitude, enabling real-time imaging of metabolic flux through key pathways such as glycolysis and oxidative phosphorylation.^14^ Unlike traditional imaging, HP-MRI may reveal early metabolic derangements, delineate hypoxic zones, track therapeutic response before anatomical change, and map segmental metabolic heterogeneity relevant to risk stratification.

This review will examine the pathophysiological basis of HCM and the principles of HP-MRI, draw on experience from oncology and cardiovascular research, and explore the potential of HP-MRI to fill the key diagnostic and management gaps that persist in HCM today (Table 1).

**Table 1.** HP-MRI Probes.

Probe	Metabolic or physiologic process highlighted
[1-^13^C]pyruvate	Glycolysis; LDH, ALT, PDH activity
[2-^13^C]pyruvate	TCA cycle metabolism
[1-^13^C]acetate	TCA cycle metabolism, fatty acid metabolism
[1-^13^C]butyrate	Fatty acid metabolism
[1-^13^C]lactate	PDH activity
^13^C urea	Perfusion
[1-^13^C]bicarbonate	pH

## Background

### Role of Sarcomere Proteins in Cardiac Function

HCM is fundamentally characterized by the dysfunction of the sarcomere. Sarcomeres represent the structural and functional units that facilitate contraction in the cardiomyocyte. Composed of interdependent protein complexes, they play a critical role in generating and modulating force.^15^ In cases of HCM, genetic mutations may occur in the genes that encode sarcomere proteins or in associated genes, thereby disrupting the effective contraction and relaxation of cardiac myocytes.^1^ This impairment initiates a cascade of pathological changes that ultimately lead to myocardial hypertrophy, fibrosis, and functional deterioration.^4^

The sarcomere comprises thick and thin filaments, specifically myosin and actin, which function in conjunction with regulatory proteins, namely troponin and tropomyosin.^16^ These components operate in a highly coordinated manner to facilitate the processes of cardiac contraction and relaxation. To date, approximately 900 mutations have been identified in the genes encoding 8 distinct sarcomere proteins, including MYH7 (β-myosin heavy chain), MYBPC3 (cardiac myosin-binding protein C), TNNT2 (cardiac troponin T), TNNI3 (cardiac troponin I), ACTC (cardiac actin), TPM1 (α-tropomyosin), MYL3 (essential myosin light chain), and MYL2 (regulatory myosin light chain). Among these mutations, those in MYH7 and MYBPC3 are the most prevalent, accounting for nearly 50% of HCM cases attributed to sarcomere mutations. In comparison, the remaining genes collectively contribute to less than 20% of cases.^17,18^ Patients are classified into 2 categories, Sarc^+^ or Sarc^−^, based on the presence of a sarcomeric mutation. Currently recognized Sarc^−^ genes implicated in HCM include CSRP3 (cysteine and glycine-rich protein 3), FHL1 (4 and a half LIM domains 1), FLNC (filamin C), FHOD3 (formin homology 2 domain-containing 3), JPH2 (junctophilin 2), PLN (phospholamban), TRIM63 (Tripartite Motif-Containing 63), and KLHL24 (Kelch-like protein 24).^19^ Regardless of the classification of Sarc^+^ or Sarc^−^, the identified mutations significantly impact sarcomere force generation, frequently leading to hypercontractility and increased energy consumption by cardiomyocytes. This hypercontractile state imposes a metabolic burden on the myocardium, initiating both compensatory and maladaptive processes that contribute to disease progression.^20-22^

### Pathophysiological Cascade

Each mutation plays a distinctive role in influencing phenotypic expression, which may subsequently affect the incidence and severity of the clinical profile, including the progression of heart failure (HF) and the risk of arrhythmogenesis. As outlined below, a range of interconnected mechanisms contribute to its clinical manifestation.^23,24^

**Increased myofilament calcium sensitivity** results from mutations in sarcomere proteins, which lead to enhanced calcium sensitivity of myofilaments.^25^ Disruptions in calcium signaling have been observed in both Sarc^−^ and Sarc^+^ HCM patients. In Sarc^+^ patients, the persistent activation of calcium-calmodulin-dependent protein kinase II (CaMKII) is understood to play a significant role in this condition.^26^ Consequently, the sarcomeres remain in a hypercontractile state characterized by elevated adenosine triphosphate (ATP) consumption, ultimately resulting in a continuous contraction state. This situation contributes to poor diastolic function of the cardiac muscle due to impaired myocardial relaxation.^27^ Altered calcium dynamics initiate myocardial and electrical remodeling at the earliest stages of the disease and are fundamental to the development of both hypertrophy and arrhythmogenesis.^28^ The hypertrophic response is further exacerbated by an increased mitochondrial workload and oxidative stress, which promote hypertrophic growth in HCM.^13^

**Hypertrophy and fibrosis** in HCM, as aforementioned, have been associated with increased calcium sensitivity. The molecular mechanisms involved align with pathways observed in other forms of cardiac hypertrophy, such as those induced by pressure overload. Key pathways affected encompass calcineurin, mitogen-activated protein kinases (MAPKs), and transforming growth factor-beta (TGF-β), in addition to non-coding RNA and various epigenetic factors.^29^ Although these molecular responses share similar characteristics, they vary depending on the underlying causal genotype. Specific pathways are hypothesized to be intricately linked to mitochondrial overload resulting from the hypercontractile state prevalent in HCM. As cardiac workload intensifies, calcium levels increase, elevating ATP consumption and stimulating mitochondria to produce additional ATP via the Krebs cycle. In the context of HCM, the myocardium exhibits an exaggerated sensitivity to calcium, necessitating an even greater supply of ATP compared to normal tissue at equivalent calcium concentrations. Nonetheless, the mitochondria within HCM cardiomyocytes often cannot meet this increased energy demand, leading to a mismatch between energy supply and demand. This imbalance results in an accumulation of adenosine diphosphate (ADP) and oxidative stress due to excess hydrogen peroxide (H_2_O_2_) within the mitochondria.^30,31^ Such oxidative stress inflicts cellular damage and serves as a significant contributor to the hypertrophy and fibrosis observed in HCM. For instance, the MAPK/ERK pathway, recognized as a pivotal driver of hypertrophic remodeling in HCM, is believed to be activated by the presence of reactive oxygen species. This pathway facilitates cardiomyocyte growth through ERK-mediated modulation of gene expression, alongside the effects of reactive oxygen species, growth factors, and mechanical stress.^32^ Hypercontractility, attributable to the heightened calcium sensitivity, further exacerbates mitochondrial workload and oxidative stress, thereby propelling the maladaptive mechanisms of hypertrophy and fibrosis.^33^ The hypertrophy typically manifests asymmetrically, with a notable preference for the basal interventricular septum.^34^ Septal hypertrophy is often associated with LVOT obstruction, which influences hemodynamics, resulting in the displacement of the mitral valve leaflets. Consequently, the mitral valve becomes increasingly prone to systolic anterior motion (SAM), which is implicated in elevated intracavitary pressures and mitral regurgitation due to impaired leaflet coaptation.^35^ In addition, hypertrophy leads to diastolic dysfunction. The resultant left atrial fibrosis, commonly triggered by hypertrophy, serves as a substrate for the development of atrial fibrillation (AF).^36^

**Coronary microvascular dysfunction** (MVD) has been associated with the primary development of HCM, frequently occurring prior to the onset of ventricular hypertrophy.^37^ The pathological alterations characteristic of MVD encompass medial hypertrophy, intimal hyperplasia, and a decrease in the luminal diameter of small intramural coronary arteries.^38^ Furthermore, the hypercontractile state of cardiomyocytes has been observed to induce pathological constriction of coronary arteries, which can chronically diminish coronary blood flow. When coupled with LVOT obstruction and increased intracavitary pressures, this process may experience further deterioration.^31^ The resultant apical ischemia and infarction can ultimately lead to the formation of left ventricular aneurysms, thereby increasing the risk of thrombus formation, HF, and fatal ventricular arrhythmias.^39^

In summary, the pathogenesis of HCM is a complex, interconnected process fundamentally driven by sarcomere dysfunction. The elevation in myofilament calcium sensitivity leads to hypercontractility, diastolic dysfunction, and an increased demand for ATP. These mechanisms subsequently result in oxidative stress and mitochondrial overload.^40^ Concurrently, signaling pathways associated with hypertrophy, such as MAPK/ERK, are activated, which promotes the growth of cardiomyocytes and the development of interstitial fibrosis.^41^ In addition, MVD exacerbates myocardial ischemia and fibrosis, contributing to progressive structural remodeling.^42^ The resultant hypertrophy, often localized to the basal interventricular septum, can cause LVOT obstruction, disrupt hemodynamics, and lead to mitral regurgitation. These alterations can further elevate intracavitary pressures and, in severe instances, may culminate in HF.^43^ Collectively, these processes establish a substrate for electrical instability, which may result in adverse clinical outcomes, including AF and ventricular arrhythmias.^44^ One of the most alarming consequences of HCM is SCD, which frequently occurs in young and often asymptomatic individuals.^45^ Therefore, it is of paramount importance to stratify patients at high risk for SCD and implement appropriate management strategies. The interrelated mechanisms at play in HCM significantly disrupt cardiac metabolism, underscoring the necessity of further investigation into cardiac metabolic processes.^46^

### Current Imaging Modalities for Cardiovascular Metabolic Analysis

A range of imaging modalities is available for the investigation of cardiac metabolism, including magnetic resonance spectroscopy (MRS), which employs both proton (^1^H) and phosphorus (^31^P) isotopes, as well as single-photon emission computed tomography (SPECT) and PET utilizing carbon-11 (^11^C) and fluorine-18 (^18^F) fatty acid tracers.^47^ However, the application of these techniques is often restricted by challenges such as low resolution and inherent technical limitations. Among these modalities, FDG-PET has emerged as a widely employed tool for metabolic imaging, exhibiting high sensitivity in evaluating myocardial viability and increasingly contributing to assessing cardiac inflammatory conditions, such as sarcoidosis.^48,49^ In the context of HCM, PET-FDG may offer valuable prognostic insights, as elevated FDG uptake has been linked to a poorer prognosis.^50^ Notably, increased FDG uptake may occur before the emergence of myocardial fibrosis, thereby indicating regions of the cardiac wall vulnerable to fibrotic transformation.^51^ Nevertheless, PET-FDG is limited in its ability to detect only areas of increased glucose uptake and does not offer insights into subsequent metabolic processes.^52^ A comprehensive understanding of downstream metabolism is essential for evaluating cardiomyocyte function, which can be effectively assessed through HP-MRI.

## Hyperpolarized MRI

### HP-MRI Technology and Principles

HP-MRI represents a potentially transformative innovation in biomedical imaging. HP-MRI utilizes dissolution dynamic nuclear polarization (dDNP) to enhance the signal of specific substrates and their metabolites by over 10 000-fold, enabling the study of real-time metabolic processes.^53^ This enhancement allows precise visualization of metabolic pathways. It has been demonstrated to provide novel diagnostic insights into cancer, neurological conditions, liver disease, and, most recently, cardiovascular conditions such as HCM.^54,55^ A broad range of substrates are available, such as [1,4-^13^C_2_]fumarate, [^13^C]urea, and [^13^C]glucose, with [1-^13^C]pyruvate being the most studied.^56^ Pyruvate is the end product of glycolysis. Depending on the body’s metabolic needs, pyruvate can be converted into either lactate (for use in anaerobic respiration) or acetyl-CoA (for use in aerobic respiration). If it is converted to acetyl-CoA, carbon dioxide or bicarbonate is released, which would be identified using HP-MRI. The distribution and conversion of pyruvate into individual metabolites, specifically lactate and bicarbonate, can provide insights into underlying biochemical processes.^57^ For example, it can detect increased lactate levels in cancer cells (known as the Warburg effect).^58^ It can also measure bicarbonate production in healthy heart tissue, showing which areas have an intact ability to undergo aerobic respiration and represent viable myocardium.^59^ In the context of neurology, HP-MRI is being increasingly studied to provide insights into neurometabolism in pathologies such as glioma, stroke, and multiple sclerosis.^60^ Advances have also been made in using HP-MRI to detect and monitor certain liver diseases and malignancies.^55^

To comprehend the mechanistic functioning of HP-MRI, it is essential to first understand the fundamental principles of MRI. MRI is predicated upon specific types of nuclei, referred to as MR-active nuclei, which possess the ability to orient themselves either in alignment with or against an external magnetic field. These nuclei feature an odd number of protons or neutrons, such as hydrogen-1, helium-3, lithium-7, or carbon-13, endowing them with the property of non-zero spin. This intrinsic spin allows for interaction with the magnetic field, resulting in the generation of a measurable signal that constitutes the foundation of MRI imaging, with the net energy difference in the magnetic state producing an MR signal.^61^ In the context of metabolic imaging involving carbon-rich molecules, such as glucose and its metabolic byproducts, carbon-13 (^13^C) is particularly noteworthy. However, owing to its low natural abundance (approximately 1.1%), ^13^C yields a relatively weak signal, insufficient for visualizing metabolic processes effectively.^62^ There are 2 principal methods to enhance the ^13^C signal: either through the enrichment of ^13^C to 99% within the relevant molecule or by augmenting the polarization level via a technique known as hyperpolarization.^63^ The most widely utilized method for achieving hyperpolarization is dDNP. In this method, a substrate, such as [1-^13^C]pyruvate, is exposed to extreme cooling in conjunction with an electron-rich compound, recognized as an electron paramagnetic agent, at temperatures approaching absolute zero (1.0-1.5 K) within a robust magnetic field ranging from 3.3 to 7 Tesla. This process is further augmented by the incorporation of a polarizing agent and the application of microwave irradiation, typically sustained for approximately 2 hours. Subsequently, the hyperpolarized substrate is swiftly dissolved into a liquid medium and administered to the subject. Once introduced in vivo, this substrate participates in metabolic reactions, thereby providing a real-time insight into significant biochemical processes, including the conversion of pyruvate to lactate, alanine, or bicarbonate. The enhanced signal from these metabolites can be detected briefly before succumbing to T1 relaxation.^64-67^

What distinguishes HP-MRI from other imaging modalities is its capacity to evaluate dynamic metabolic fluxes without the use of ionizing radiation. This characteristic provides a significant advantage over FDG-PET, which relies on ionizing radiation and primarily reveals areas of increased glucose uptake, lacking insights into downstream metabolic byproducts.^52^ By addressing these limitations and complementing traditional structural imaging techniques, HP-MRI effectively fills critical gaps in metabolic imaging. HP-MRI has been extensively investigated for applications in cancer imaging, an area we will further explore to establish a foundation for discussing its relevance in the context of HCM.

### Applications of HP-MRI in Cancer Imaging

HP-MRI first found clinical traction in oncology because malignant cells re-wire metabolism long before they become anatomically conspicuous. A hallmark is the Warburg effect: tumors favor glycolysis and lactate production even in the presence of oxygen. When hyper-polarized [1-^13^C]pyruvate is injected, rapid conversion to ^13^C-lactate via lactate dehydrogenase (LDH) serves as a real-time measure of this shift.

In a Myc-driven liver-cancer model, a rising lactate-to-pyruvate ratio appeared weeks before MRI could detect a mass, flagging transformation at a “metabolic pre-clinical” stage.^68^ Similar findings were reported in high-risk pancreatic cysts, where metabolic HP-MRI is under prospective evaluation.^69^ These data parallel HCM, in which metabolic changes precede hypertrophy. Additionally, Oxidative decarboxylation of pyruvate yields ^13^C-bicarbonate, so the lactate : bicarbonate ratio simultaneously gages glycolytic versus oxidative metabolism. In human prostate cancer (2013 first-in-man study) and glioma cohorts, regions with suppressed bicarbonate signal correlate with hypoxia and poor prognosis.^70^ The same metric could delineate hypoxic or energy-starved zones in hypertrophic myocardium.

Multi-center studies have shown that higher-grade lesions in prostate, kidney, breast and brain cancers exhibit stronger hyper-polarized lactate signals, reflecting elevated LDH and monocarboxylate-transporter-4 expression.^61,67,71-74^ For example, HP-MRI detected high lactate in aggressive renal-cell carcinoma that appeared indolent on conventional MRI.^61^ This principle underscores HP-MRI’s capacity to stratify disease severity, an analog to distinguishing low-risk from arrhythmia-prone HCM phenotypes. Furthermore, a fall in lactate or rise in bicarbonate within days of chemotherapy predicts tumor shrinkage weeks later on anatomical imaging. Breast-cancer patients receiving neoadjuvant doxorubicin showed a ⩾30% drop in lactate signal after the first cycle, accurately forecasting pathological response.^74^ Similarly HP-MRI could provide early feedback in HCM patients started on myosin inhibitors or metabolic modulators, well before ventricular geometry changes. Multi-slice HP-MRI reveals that a single tumor often contains lobes with markedly different lactate signals. This heterogeneity mirrors preliminary human HCM data in which segmental lactate-to-bicarbonate ratios vary across the left ventricle. Mapping such metabolic regions may be helpful in HCM patients in terms of risk stratification.

Key mechanistic parallels to HCM are summarized as a list below. Both tumors and hypertrophic cardiomyocytes:

- up-regulate LDH;
- uncouple glycolysis from oxidative phosphorylation;
- generate regions of relative hypoxia despite adequate macro-perfusion.

In summary, the cancer literature establishes HP-MRI as a safe, non-ionizing tool for tracking in-vivo metabolic flux. The same core metrics, pyruvate utilization, lactate surge, and bicarbonate suppression, are directly translatable to the metabolic derangements that drive HCM pathophysiology.

### HP-MRI and Metabolism in the Normal Heart

**Figure 1.**
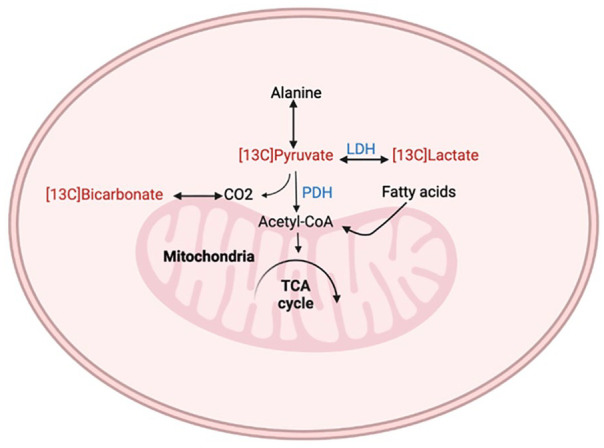
Key metabolic pathways highlighted by HP-MRI relevant to HCM. Metabolites labeled in red provide key insights—enzymes labeled in blue are key regulators of these metabolic pathways.

The application of HP-MRI for HCM is fundamentally rooted in the metabolic pathways of the myocardium. The heart demonstrates the highest ATP consumption among all organs. It primarily fulfills its energy requirements through the oxidation of fatty acids, which accounts for 60% to 90% of its energy source, with the balance derived from pyruvate oxidation (20%-30%) and glycolysis (less than 10%).^75^ Consequently, the principal metabolic pathways are the oxidation of fatty acids and pyruvate. Both pathways necessitate the conversion of their respective substrates to acetyl-CoA prior to oxidation. Pyruvate is converted to acetyl-CoA via the activity of the pyruvate dehydrogenase (PDH) enzyme complex, while fatty acids are metabolized to acetyl-CoA through mitochondrial β-oxidation. Various regulatory mechanisms favor fatty acid oxidation over glucose oxidation, with the PDH complex serving as a critical determinant in this process. This includes the phosphorylation-inhibition of PDH and end-product inhibition of PDH by acetyl-CoA and NADH.^76^ During periods of heightened energy demand, such as during aerobic exercise or postprandial states, the heart demonstrates a propensity to utilize glucose and other carbohydrates more significantly.^75^ This phenomenon is referred to as metabolic flexibility, which is a vital characteristic of a healthy heart in meeting elevated ATP requirements. It is posited that the lower ATP/O2 ratio associated with fatty acid oxidation necessitates greater oxygen consumption to generate ATP compared to glucose oxidation.^77^ Accordingly, under conditions of increased cardiac workload, where oxygen availability may be compromised, a shift toward glucose oxidation is observed. This review will examine how HP-MRI can elucidate these metabolic interactions and provide valuable insights into cardiac metabolism. Pyruvate is pivotal in energy generation within the heart and has emerged as the primary molecule investigated in the context of HP-MRI as a cardiac imaging modality. Labeled ^13^C pyruvate can traverse 3 main metabolic pathways: conversion to [1-^13^C]alanine via the action of alanine aminotransferase (AST), conversion to [1-^13^C]lactate via lactate dehydrogenase (LDH), or conversion to acetyl-CoA with [^13^C]bicarbonate produced as a by-product via PDH.^56^ The metabolic flux through PDH is of significant interest, as it is a key enzyme influencing metabolic pathways; the production of [^13^C]bicarbonate allows for measurement of this flux. Additionally, an increased production of [1-^13^C]lactate signals a diversion from oxidative metabolism toward glycolysis, indicative of hypoxic conditions and potentially reflecting regions of ischemia. Figure 1 summarizes the key metabolic pathways assessed by [1-^13^C]pyruvate relevant to HCM.

A targeted, non-systematic search was conducted using PubMed, Scopus, and Google Scholar to identify studies on HP-MRI in HCM. Search terms included combinations of “hyperpolarized MRI,” “^13^C pyruvate,” “cardiac metabolism.” Both preclinical and clinical studies were considered. Inclusion criteria focused on studies involving HP-MRI applications in cardiac tissues, while papers without cardiac relevance or not involving hyperpolarized imaging were excluded. The search included articles published in English from 2000 to May 2024.

HP-MRI has previously been applied in human studies across a range of cardiac conditions, including healthy hearts, diabetic cardiomyopathy, coronary artery disease, and HCM. Numerous studies have also explored its use in diverse animal models to investigate metabolic flux and pathophysiological changes. Tables 2 and 3 provide a comprehensive overview of these studies. Reference lists of relevant reviews and key papers were also screened for additional sources.

**Table 2.** Human Studies Utilizing HP-MRI.

Author	Year	Participants	Age	Gender	Substrate	Outcomes
Sinha et al^78^	2024	5 HCM patients, 7 healthy volunteers.	HCM patients: 40 ± 8 y, healthy volunteers: 34 ± 8 y.	Not specified.	[1-^13^C]pyruvate.	kPB (pyruvate-to-bicarbonate conversion) and kPL (pyruvate-to-lactate conversion) were assessed using HP MRI. HCM patients showed varied metabolic phenotypes, including uncoupling of glycolysis and glucose oxidation and altered kPB/kPL dynamics. Healthy volunteers demonstrated significant metabolic flexibility with kPB correlating strongly with blood glucose. Results suggest potential genotype-specific metabolic patterns in HCM patients.
Joergensen et al^79^	2024	18 participants (12 HF patients, 6 healthy controls)	Mean: 58 y	10 male, 8 female	[1-^13^C]pyruvate	Correlated metabolite ratios (lactate/bicarbonate) with left ventricular function, enabling metabolic phenotyping for HF.
Larson et al^59^	2023	7 healthy volunteers	25-44 y	5 male, 2 female	[1-^13^C]pyruvate	Demonstrated regional metabolic mapping and changes in PDH/LDH activity in response to glucose, showing potential for detecting substrate-level metabolic shifts.
Joergensen et al^80^	2022	6 healthy human participants	Mean: 34 y	3 male, 3 female	[1-^13^C]pyruvate	Demonstrated increased PDH flux and lactate production during adenosine stress, showcasing metabolic flux assessment under stress.
Apps et al^81^	2021	2 patients with myocardial infarction	Case 1: 67 y; Case 2: 76 y	Case 1: Male; Case 2: Female	[1-^13^C]pyruvate	Case 1 (Non-ST segment elevation myocardial infarction): Preserved ^13^C-bicarbonate and [1-^13^C]lactate signals in viable subendocardial infarction regions; absence of signals in nonviable transmural infarction areas. Case 2 (ST segment elevation myocardial infarction): Similar pattern with preserved signals in viable regions and absence in nonviable areas. Demonstrated feasibility of hyperpolarized ^13^C imaging for assessing oxidative metabolism and viability in ischemic myocardium. Highlights potential for stratifying patients for revascularization based on metabolic imaging
Rider et al^82^	2020	13 T2DM patients and 12 healthy controls	Mean: 54 y	7 male, 6 female in T2DM; 6 male, 6 female in control	[1-^13^C]pyruvate	Demonstrated reduced PDH flux in T2DM patients compared to controls and showed changes in metabolic flux after glucose challenge.
Park et al^72^	2020	9 women with biopsy-proven breast cancer requiring neoadjuvant doxorubicin (initial enrollment of 10; 1 excluded due to technical issues)	47 ± 5 y	Female	[1-^13^C]pyruvate.	Doxorubicin treatment was associated with a significant decrease in hyperpolarized [^13^C]bicarbonate/total carbon signal, suggesting subtle mitochondrial injury. No significant changes were observed in [1-^13^C]lactate or [1-^13^C]alanine levels post-treatment. Deterioration in left ventricular global longitudinal strain (LVGLS) was noted, with minor changes in plasma hemoglobin and hs-cTnT levels. The study demonstrated the feasibility and reproducibility of HP-MRI for detecting early myocardial metabolic changes.
Cunningham et al^83^	2016	Healthy volunteers (n = 4)	Mean: 41 y (range 28-48)	Male	[1-^13^C]pyruvate	Showed feasibility of human cardiac ^13^C metabolic imaging, detecting pyruvate flux and lactate production in the myocardium.

**Table 3.** Animal Studies Utilizing HP-MRI.

Author	Year	Participants	Substrate	Outcomes
Lewis et al^84^	2024	Male long Evans rats	[1-^13^C]pyruvate, [2-^13^C]pyruvate	Demonstrated reduced PDH flux and altered TCA cycle metabolism in obesity, reversible with caloric restriction or liraglutide.
Shaul et al^85^	2023	Male mouse hearts (n = 30)	[1-^13^C]pyruvate	Intracellular acidification reduced PDH and LDH activities, modulated by Na+/H+ exchanger inhibition.
Fuetterer et al^86^	2022	Pigs (n = 8, 5 infarction, 3 control)	[1-^13^C]pyruvate, [2-^13^C]pyruvate	Demonstrated correlation of lactate-to-bicarbonate ratio changes with myocardial recovery post-infarction, revealing dynamic metabolic and structural changes.
Tougaard et al^87^	2021	Rats (n = 35)	[1-^13^C]pyruvate	β-blockers improved heart function; metabolic flux showed variability.
Savic et al^88^	2021	Diabetic and control rats (n = 36)	[1-^13^C]pyruvate	Meldonium improved PDH flux and post-ischemic recovery in diabetic hearts.
Abdurrachim et al^89^	2019	SHHF rats and controls	[3-^13^C]acetoacetate, [1-^13^C]pyruvate	Empagliflozin reduced ketone utilization in diabetic hearts while maintaining glucose metabolism.
Le Page et al^90^	2019	Male Wistar rats (n = 3 groups)	[1-^13^C]pyruvate	Acute hypoxia reduced PDH flux; chronic hypoxia normalized metabolism to normoxic levels.
Tougaard et al^91^	2018	Pigs (n = 5)	[1-^13^C]pyruvate	Demonstrated acute hypertensive stress leading to altered cardiac metabolism.
Tougaard et al^92^	2018	Pigs (n = 18, 13 fasted, 5 fed)	[1-^13^C]pyruvate	Fed state showed increased lactate/pyruvate ratios, correlating with reduced free fatty acid levels.
Wespi et al^93^	2018	Rats (n = 6)	[1-^13^C]pyruvate	Liver metabolism overestimates cardiac lactate production in spectroscopy; imaging preferred.
Miller et al^94^	2018	Isolated rat hearts	[1,4-^13^C_2_]fumarate	Malate production effectively indicated necrosis following myocardial infarction.
Rohm et al^95^	2018	Mouse model (bV59M mice)	[1-^13^C]pyruvate, [2-^13^C]pyruvate	Hyperglycemia reduced cardiac output, stroke volume, and PDH flux. Euglycemia restored cardiac metabolism and function.
Hansen et al^96^	2017	Pigs (n = 4)	[1-^13^C]pyruvate	Showed that GIK infusion modulates cardiac substrate metabolism, increasing pyruvate to its derivatives.
Steinhauser et al^97^	2017	Rats	[1-^13^C]pyruvate	Isoflurane altered cardiac metabolism, reducing bicarbonate and increasing lactate production.
Mariotti et al^98^	2016	Crystalloid-perfused rat hearts	[1-^13^C]pyruvate	Identified non-linear pyruvate-bicarbonate kinetics linked to NADH availability during pyruvate infusion.
Chen et al^99^	2015	8 pigs (5 infarction, 3 controls) monitored over 9 wk post-infarction	[1-^13^C]pyruvate	Identified metabolic changes in infarcted myocardium (lactate/bicarbonate ratios), correlating with structural recovery and contractility improvements.
Seymour et al^100^	2015	Sprague Dawley rats with aortic banding and controls	[1-^13^C]pyruvate	Highlighted enhanced glycolysis in hypertrophied hearts, with no significant change in glucose oxidation.
Dodd et al^101^	2014	Female Wistar rats post-MI	[1-^13^C]pyruvate and [2-^13^C]pyruvate	Observed impaired Krebs cycle activity in MI hearts, with progressive metabolic dysfunction correlating with cardiac impairment.
Khemtong et al^102^	2014	Perfused rat hearts	[1-^13^C]pyruvate	Detected rapid changes in lactate after adrenergic stimulation, reflecting altered glycogen metabolism.
Aquaro et al^103^	2013	7 male pigs with induced ischemia-reperfusion using a pneumatic coronary occluder	[1-^13^C]pyruvate	Detected regional metabolic changes (lactate/bicarbonate) and spatial resolution in ischemia and reperfusion.
Lauritzen et al^104^	2013	Fasted rats	[1-^13^C]pyruvate	High-dose GIK infusion significantly increased bicarbonate signals, suggesting improved myocardial glucose oxidation.
Giovannetti et al^105^	2013	Pigs	[1-^13^C]pyruvate	Demonstrated the effectiveness of a novel RF coil system in mapping metabolites distribution in pig heart models.
Menichetti et al^106^	2012	Anesthetized pigs	[1-^13^C]pyruvate	Observed increased conversion rates of pyruvate to bicarbonate and lactate under dobutamine stress, linking changes to cardiac oxygen consumption.
Flori et al^107^	2012	Pigs	[1-^13^C]pyruvate	Showed large-dose hyperpolarized pyruvate imaging is feasible for detecting cardiac metabolism and optimized protocols for large animal models.
Schroeder et al^108^	2012	Pigs with dilated cardiomyopathy (n = 5)	[2-^13^C]pyruvate	Early decrease in cardiac metabolism and bicarbonate production correlating with HF progression
Frijia et al^109^	2011	3 mini-pigs	[1-^13^C]pyruvate	Demonstrated spatial localization of pyruvate and its metabolites in the heart using 3D imaging.
Santarelli et al^110^	2011	Medium-sized animals	[1-^13^C]pyruvate	Emphasized the importance of SNR on quantification of cardiac metabolism and highlighted the optimization of experimental conditions.
Schroeder et al^111^	2011	Fed and fasted rats	[1-^13^C]pyruvate	Found differential PDH activity regulation depending on fed or fasted state, suggesting feedback inhibition by acetyl-CoA.
Menichetti et al^112^	2010	4 mini-pigs	[1-^13^C]pyruvate	Demonstrated feasibility of in vivo cardiac metabolism imaging using hyperpolarized pyruvate in pigs, with real-time tracking of lactate, alanine, and bicarbonate production.
Moreno et al^113^	2010	Isolated rat hearts	[1-^13^C]pyruvate	Demonstrated sensitivity of HP pyruvate metabolism to competing substrates, requiring specific concentrations for effective cardiac metabolism measurement.
Golman et al^114^	2008	Male pigs (n = 6)	[1-^13^C]pyruvate	Feasibility demonstrated for detecting metabolic changes during ischemic episodes.
Tyler et al^115^	2008	Mice	[1-^13^C]pyruvate, [2-^13^C]pyruvate	Demonstrated real-time visualization of PDH and LDH activities, highlighting pyruvate as a metabolic marker for ischemic and infarcted tissue.

### HP-MRI and HCM

In HCM, the myocardium experiences considerable metabolic alterations driven by increased energy demands and sarcomere dysfunction. Under normal circumstances, the heart predominantly utilizes fatty acid oxidation for its energy requirements. However, in the context of HCM, there is a shift toward enhanced glucose utilization, which occurs due to mitochondrial dysfunction and impaired oxidative metabolism.^13^ This transition creates a discordance between the rates of glycolysis and glucose oxidation, resulting in excessive lactate production and suboptimal ATP generation. The hypercontractile state of HCM cardiomyocytes further amplifies ATP demand, thereby exacerbating energy deficiencies and oxidative stress.^30^ Over time, these metabolic disruptions contribute to structural changes such as hypertrophy, fibrosis, and myocyte dysfunction, which complicate the clinical presentation of HCM and may lead to diastolic dysfunction, arrhythmias, and myocardial ischemia.^24^ Given that these metabolic changes precede subsequent structural alterations and clinical manifestations, HP-MRI has the potential to identify HCM at an early stage, prior to the emergence of clinical symptoms.

A study by Sinha et al focuses on abnormal metabolic processes in patients with HCM utilizing hyperpolarized [1-^13^C]-pyruvate MRI.^78^ This investigation compares cardiac metabolic pathways in 7 healthy individuals and 5 HCM patients. The findings indicate that healthy subjects consistently exhibit significant metabolic flexibility, characterized by increased bicarbonate production following oral glucose intake. In contrast, HCM patients displayed heterogeneous metabolic phenotypes. Specifically, 2 HCM patients demonstrated elevated lactate production and average bicarbonate production in response to oral glucose, suggesting upregulation of GLUT1 and LDHA activity, likely due to an uncoupling of glycolysis and glucose oxidation. Furthermore, 2 HCM patients exhibited elevated bicarbonate and lactate production even during fasting, which may indicate heightened energy demands. The final HCM patient presented with reduced bicarbonate production in both fasting and fed states, potentially signaling an uncoupling of glycolysis and glucose oxidation. The variability in metabolic phenotypes among HCM patients may imply a potential link to diverse genotypes of HCM. These findings are promising and suggest the potential role of HP-MRI in managing HCM.

Numerous studies have highlighted the extensive application of HP-MRI in evaluating human cardiac metabolism across diverse physiological scenarios (Table 2). Cunningham et al initially demonstrated the feasibility of cardiac HP-MRI by successfully detecting pyruvate flux and lactate production within the myocardium.^83^ In healthy subjects, Joergensen et al reported increased PDH flux and lactate production during an adenosine stress test when compared to resting cardiomyocytes.^80^ Moreover, Larson et al elucidated metabolic adaptations in response to glucose, examining the production of ^13^C-lactate and ^13^C-bicarbonate. They observed a significant correlation between elevated ^13^C-bicarbonate levels and increased glucose concentrations, reflecting enhanced PDH flux. Furthermore, their research showcased regional metabolic mapping, revealing that the left ventricle exhibited the most pronounced increase in ^13^C-bicarbonate levels in the fed state relative to fasting conditions.^59^ Rider et al underscored diminished PDH flux following an oral glucose challenge in patients with type 2 diabetes mellitus (T2DM) compared to healthy controls.^82^ Joergensen et al investigated patients suffering from ischemic heart disease and dilated cardiomyopathy characterized by reduced left ventricular ejection fractions (LVEF).^79^ Their findings indicated a strong correlation between metabolite ratios and left ventricular function in HF, with a high lactate signal being associated with poor contractility and a markedly low bicarbonate signal observed in cases where LVEF was less than 30%. In addition, Apps et al illustrated the utility of HP-MRI in the context of myocardial infarction, primarily based on the preserved ^13^C-bicarbonate signal’s ability to differentiate between viable and nonviable myocardium, which signifies areas of oxidative metabolism and serves as a hallmark of tissue viability.^81^ Furthermore, Park et al identified subtle myocardial mitochondrial injury in breast cancer patients undergoing doxorubicin treatment, as evidenced by a decline in [^13^C]bicarbonate levels.^72^

Research utilizing hyperpolarized [1-^13^C]pyruvate has been conducted across various animal models, with the findings summarized in Table 3. These investigations confirm the efficacy of HP-MRI in detecting metabolic alterations during ischemia-reperfusion, hypertrophy, and infarction, as well as in response to stress conditions such as hypoxia or adrenergic stimulation. Notable discoveries include PDH and lactate dehydrogenase (LDH) activity variations, while lactate-to-bicarbonate ratios provide insights into oxidative metabolism and glycolysis. For instance, ischemic cardiac tissues exhibited diminished PDH activity and modified metabolite ratios. Interventions such as meldonium, glucose-insulin-potassium (GIK) therapy, and β-blockers were shown to enhance PDH flux, thereby facilitating increased myocardial glucose oxidation and consequent recovery. Investigations involving diabetic and hypertrophic cardiac models revealed reduced PDH activity and metabolic inflexibility; however, improvements in metabolic flexibility and PDH flux were noted following targeted interventions. Collectively, the outcomes of both animal and human studies highlight the promise of HP-MRI as a versatile, innovative, and well-tolerated technology for evaluating metabolic flux in cardiomyocytes within both healthy and diseased populations.

## Discussion

### Clinical Relevance of Metabolic Flexibility in HCM

HP-MRI represents a promising and innovative imaging modality for the diagnosis and monitoring of HCM. This technique offers a unique capability to directly evaluate metabolic pathways, positioning it as a potentially transformative tool in the clinical management of HCM. A central pathogenic characteristic of HCM is the hypercontractile state of cardiomyocytes, which arises from augmented calcium sensitivity attributable to various mechanisms.^37,41^ Consistently, research has indicated impaired myocardial relaxation as an early hallmark feature of HCM, preceding the development of hypertrophy.^1^ Additionally, animal and human studies have documented alterations in cellular metabolism and energy production before any observable cardiac hypertrophy. This is evident in symptomatic and asymptomatic patients, who display diminished cardiac efficiency, underscoring the excessive ATP consumption required to maintain increased contractility.^116^ This condition is hypothesized to contribute to mitochondrial energy burden and disrupt normal energy utilization. More significantly, the disturbances pertinent to HP-MRI involve the metabolic adaptations that indicate a shift toward pyruvate oxidation and away from fatty acid metabolism, a mechanism particularly pronounced in HCM due to elevated energy demands.^13^ Furthermore, histological evaluations of HCM biopsies reveal a reduction in the expression of proteins responsible for fatty acid oxidation. Corroborating evidence has emerged from studies that indicate decreased expression of fatty acid receptors.^117^ For instance, CD36, a receptor vital for the absorption of fatty acids, which serve as the primary energy source for the heart, has been shown to be downregulated in symptomatic cases of HCM, while remaining unaffected in asymptomatic cases.^118,119^

Microvascular dysfunction is frequently observed prior to the appearance of structural abnormalities in the heart, potentially impairing oxygen availability and thereby affecting cardiomyocyte metabolism. FDG-PET is the sole clinically available modality capable of assessing myocardial metabolism, though it is limited to quantifying glucose uptake without providing information on downstream metabolites. Consequently, HP-MRI, with its ability to detect downstream metabolic alterations that often manifest before structural changes, could be instrumental in the early diagnosis of HCM. This process may be especially valuable for individuals genetically predisposed to HCM, as early detection could facilitate timely and effective management strategies, potentially slowing disease progression.

### Link Between Metabolic Phenotypes and HCM Genotypes

Metabolic adaptability has been identified as distinct between Sarc^+^ and Sarc^−^ patients. Nollet et al reported that remodeling in Sarc^−^ HCM is more closely associated with mitochondrial dysfunction and a reduction in acylcarnitine when compared to Sarc^+^ HCM.^120^ Acylcarnitine functions as a transporter molecule, facilitating the movement of long-chain fatty acids from the cytosol to the mitochondria for the purpose of fatty acid oxidation. Furthermore, the study indicated a decrease in metabolites associated with glucose oxidation, a finding that aligns with the consequences of mitochondrial dysfunction attributable to disrupted bioenergetic homeostasis. In contrast, Sarc^+^ remodeling is characterized by increased biosynthetic metabolites, likely to support cardiac hypertrophy. While the authors acknowledge the necessity for additional research to comprehensively examine how the downregulation of biosynthetic processes in Sarc^−^ patients contributes to disease progression, their findings present promising implications for HP-MRI. Numerous studies have indicated that Sarc^+^ patients experience significantly earlier onset of clinical events and a higher incidence of adverse outcomes, facing at least twice the risk of mortality.^121^ Conversely, Sarc^−^ patients devoid of a family history of HCM exhibit a mortality rate comparable to that of the general population, suggesting that distinguishing between Sarc^−^ and Sarc^+^ HCM could be advantageous. Although certain patterns may be observed in CMR and echocardiography that differentiate Sarc^−^ from Sarc^+^ presentations, these indicators do not definitively ascertain the genotype of HCM.^122^ HP-MRI possesses the potential to play a pivotal role in distinguishing between the 2 genotypic forms of HCM in the early stages of disease progression, thereby possibly informing management strategies and prognostic assessments. Nevertheless, conflicting evidence exists regarding the differences in metabolic adaptability between Sarc^−^ and Sarc^+^ patients. For instance, Ranjbarvaziri et al found no significant disparity in mitochondrial function and related molecules between these patient groups. This underscores the pressing need for larger cohort studies and comprehensive reviews to achieve definitive conclusions.

Furthermore, various sarcomere mutations, including MYH7, MYBPC3, TNNT2, and TNNI3, may exhibit unique characteristics within their metabolic pathways. These distinct metabolic pathways could be evaluated through HP-MRI and potentially aid in identifying the specific mutation present. The different mutations are reportedly associated with unique phenotypic manifestations. For instance, MYH7 mutations have been correlated with a higher incidence of atrial fibrillation, ventricular arrhythmias, and an earlier age of onset, among other characteristics.^1^ In contrast, TNNT2 mutations are associated with milder and more atypical hypertrophy, increased left ventricular fibrosis, and a greater incidence of HF, along with other notable features.^123^ Given that HCM mutations display distinct features, early identification of these mutations could facilitate the prompt implementation of tailored management strategies, potentially preventing disease progression and associated complications. However, current literature offers limited insights regarding metabolic pathways across different mutations. Consequently, further research is necessary to ascertain whether sufficiently significant differences exist to render HP-MRI a valuable tool in this context.

### Differentiating HCM From Other Conditions

Several disorders exhibit clinical presentations similar to HCM, making it challenging to differentiate from HCM. These conditions, referred to as HCM phenocopies, can be categorized based on their underlying etiologies, which include glycogen storage disorders, lysosomal storage disorders, cardiac amyloidosis, athlete’s heart, mitochondrial cytopathies, and hypertensive heart disease.^20,124^ Distinct features may assist clinicians in considering investigations for HCM phenocopies. CMR is particularly valuable for detecting subtle structural differences between HCM and its phenocopies. However, various conditions frequently present overlapping imaging findings, such as patterns of hypertrophy and LGE.^125^ Such that there may be a considerable delay in achieving accurate diagnoses. More importantly, there may be a likelihood that these disorders are frequently misidentified as HCM. Given that the management and clinical trajectories of HCM phenocopies diverge considerably from those of HCM, it is imperative to enhance the diagnostic methodologies. The potential of HP-MRI to detect unique metabolic profiles may play a pivotal role in improving the diagnosis of HCM. Further research examining metabolic pathways in HCM versus HCM phenocopies utilizing HP-MRI is essential to evaluate its effectiveness in this context thoroughly.

### Potential Role of HP-MRI Guiding Treatment, Risk Stratification, and Prognosis

There is a paradigm shift toward personalized treatment of HCM, focusing on preventing SCD while reducing the occurrence of HF and arrhythmias. Emerging treatment options that change the metabolic profile, such as myosin inhibitors, demonstrate that HCM is impacted on a cellular level. Other treatment strategies, namely targeting mitochondrial function, have been proposed, which aim to restore metabolic pathways to their normative state, offering a potential for HP-MRI in evaluating therapeutic outcomes on a cellular level.^126^ Joergensan et al illustrated the utility of [1-^13^C]pyruvate HP-MRI in distinguishing metabolic phenotypes in patients diagnosed with HF. Their findings revealed a robust correlation between baseline lactate and bicarbonate ratios and the improvement in LVEF at follow-up in patients with ischemic heart disease, while no such correlation was observed in those without ischemic heart disease. This underscores the necessity for further research on HCM and HP-MRI across various contexts, particularly regarding responses to specific therapeutic interventions. The efficacy of HP-MRI in monitoring therapeutic responses has been firmly established through extensive studies in cancer treatments; however, additional investigations are warranted within the realm of HCM.

Lactate to bicarbonate ratio could serve as biomarkers for disease severity and progression. This is based on the principle of a mismatch between energy requirement and energy production, which can be triggered by myocardial ischemia, HF, and HCM, among other conditions. In HCM, hypercontractility of cardiomyocytes, MVD, and progression to HF or LVOT obstruction can all result in increased energy requirement. Studies in animal models have consistently demonstrated an increase in anaerobic glycolysis in myocardial ischemia and, as a result, a rise in lactate production. Lactate to bicarbonate production can be used to assess anaerobic respiration versus aerobic respiration, showing the severity of the disease. Hence, by assessing bicarbonate and lactate progression, it may be possible to assess the degree of disease severity and, hence, aid in stratifying patients by risk. For example, in LVOT obstruction, the cardiomyocytes would likely be under more burden to contract harder due to increased afterload. As a result, cardiomyocytes would likely utilize more anaerobic respiration, resulting in an altered lactate:bicarbonate ratio and, hence, increased disease severity. The pathophysiology behind SCD in HCM is not fully understood but has been linked to increased myofilament calcium sensitivity in mice studies.^127^ As an increased calcium sensitivity can be reflected by an increase in energy demands, HP-MRI has the potential to play a significant role in stratifying patients at risk of SCD beyond LGE.

## Current Limitations and Future Opportunities

### Conceptual and Research Limitations

HP-MRI offers an exciting new avenue for early diagnosis and advanced management of HCM. Although research specifically focusing on HCM and HP-MRI is limited, the findings from HP-MRI applications in oncology are highly promising. Naturally, as detailed below, there are many challenges associated with this technology.

As surmised in the review, only 1 study has directly investigated the use of HP-MRI in HCM, which involved 5 HCM patients and 7 controls. Although the findings were encouraging, demonstrating distinct metabolic profiles in HCM patients, the small sample size significantly limits the generalizability of the results, and the conclusions drawn remain largely theoretical at this stage. Moreover, much of the supporting evidence is extrapolated from other disease models, which may not fully replicate the unique pathophysiological features of HCM, posing additional translational challenges. Therefore, further investigations involving larger cohorts with longer longitudinal follow-ups are warranted. More importantly, future research should emphasize the effectiveness of HP-MRI in the early diagnosis of HCM, particularly in young patients with a familial history. This approach could enhance our understanding of whether the metabolic alterations that precede structural changes in HCM may serve as valid diagnostic indicators. Furthermore, HP-MRI could prioritize the characterization and correlation of specific genotypes with their corresponding metabolic phenotypes. The utility of the new myosin inhibitors may also have implications for metabolic pathways. Additional research is necessary to explore the utility of HP-MRI as a tool for monitoring therapeutic responses to novel HCM treatments, such as myosin inhibitors. Although a research tool, it would be beneficial to integrate complementary diagnostic modalities, such as echocardiography, CMR, and genetic testing, that would facilitate a more comprehensive understanding of the disease trajectory in individual HCM patients.

### Practical and Implementation Barriers

Despite its promising capabilities, the widespread adoption of HP-MRI in clinical practice faces significant implementation barriers. A primary technical limitation is the short half-life of hyperpolarized ^13^C substrates, typically around 2 minutes, which constrains the imaging window and necessitates precise coordination of substrate delivery, image acquisition, and metabolic activity. Although recent advances in pulse sequences and imaging protocols have improved signal efficiency, the challenge of acquiring high-quality, reproducible data within this narrow timeframe remains substantial.

In addition to the imaging constraints, HP-MRI requires highly specialized infrastructure. dDNP equipment necessary for generating hyperpolarized substrates is not available in most clinical imaging centers, and its acquisition and maintenance involve considerable cost. Furthermore, the technique demands a multidisciplinary team with expertise in radiochemistry, MR physics, and metabolic imaging, limiting its scalability in standard hospital settings. These constraints also reduce patient throughput, as each scan requires substantial preparation time and resources. While alternative hyperpolarization methods, such as parahydrogen-induced polarization (PHIP), offer potential for faster and more cost-effective implementation, they remain in early experimental stages. Addressing these barriers through technological innovation, workforce training, and streamlined protocols will be essential for transitioning HP-MRI from a research tool into routine clinical application.

## Conclusion

The pathophysiology of HCM is characterized by sarcomere dysfunction, hypercontractility, and abnormal energy metabolism, which contribute to maladaptive remodeling, resulting in myocyte hypertrophy and fibrosis. Traditional imaging techniques, such as echocardiography and CMR, provide critical insights into structural abnormalities; however, they do not illuminate metabolic pathways. Although FDG-PET offers metabolic insights, its focus is limited to glucose uptake and lacks comprehensive downstream analysis. In contrast, HP-MRI facilitates the examination of these downstream metabolic processes. More importantly, it represents a novel approach to imaging HCM by elucidating distinct metabolic pathways. Utilizing hyperpolarized substrates such as [1-^13^C]pyruvate and measuring its downstream metabolites, lactate, and bicarbonate, HP-MRI can yield essential information regarding disease severity and progression. This review highlights the potential of HP-MRI in identifying diverse HCM phenotypes, differentiating them from phenocopies, and evaluating therapeutic responses. Despite the present limitations in research, technical challenges, and the slow adoption of HP-MRI in clinical practice, this method holds considerable promise. The integration of HP-MRI with conventional imaging modalities could significantly enhance the management of HCM. To validate its clinical efficacy, further large-scale, longitudinal studies are needed.
